# Association between multimorbidity patterns and disability among older people covered by long-term care insurance in Shanghai, China

**DOI:** 10.1186/s12889-021-10463-y

**Published:** 2021-02-27

**Authors:** Zijing Wang, Wenjia Peng, Mengying Li, Xinghui Li, Tingting Yang, Cancan Li, Huosheng Yan, Xianjie Jia, Zhi Hu, Ying Wang

**Affiliations:** 1 School of Health Service Management, Anhui Medical University, Hefei, 230032 China; 2 Epidemiology and Health Statistics, School of Public Health, Bengbu Medical College, Bengbu, Anhui China; 3 School of Public Health, Key Laboratory of Public Health Safety, NHC Key Laboratory of Health Technology Assessment, Fudan University, Shanghai, 200032 China

**Keywords:** Multimorbidity, Chronic disease, Disability, Older people

## Abstract

**Background:**

Functional disability and multimorbidity are common among older people. However, little is known about the relationship between functional disability and different multimorbidity combinations. We aimed to identify multimorbidity patterns and explore the associations between these patterns and functional disability.

**Methods:**

We investigated a multi-stage random sample of 1871 participants aged ≥60 years and covered by long-term care insurance in Shanghai, China. Multimorbidity was defined as the simultaneous presence of two or more chronic diseases in an individual. Participants completed scales to assess basic and instrumental activities of daily living (BADL and IADL, respectively). Multimorbidity patterns were identified via exploratory factor analysis. Binary logistic regression models were used to determine adjusted associations between functional disability and number and patterns of multimorbidity.

**Results:**

Multimorbidity was present in 74.3% of participants. The prevalence of BADL disability was 50.7% and that of IADL disability was 90.7%. There was a strong association between multimorbidity and disability. We identified three multimorbidity patterns: musculoskeletal, cardio-metabolic, and mental-degenerative diseases. The cardio-metabolic disease pattern was associated with both BADL (OR 1.28, 95%CI 1.16–1.41) and IADL (OR 1.41, 95%CI 1.19–1.68) disability. The mental-degenerative disease pattern was associated with BADL disability (OR 1.55, 95%CI 1.40–1.72).

**Conclusions:**

Multimorbidity and functional disability are highly prevalent among older people covered by long-term care insurance in Shanghai, and distinct multimorbidity patterns are differentially associated with functional disability. Appropriate long-term healthcare and prevention strategies for older people may help reduce multimorbidity, maintain functional ability, and improve health-related quality of life.

**Supplementary Information:**

The online version contains supplementary material available at 10.1186/s12889-021-10463-y.

## Background

China has a rapidly aging population. By 2050, it is estimated that 400 million people will be aged over 65 years, and 150 million will be aged over 80 years [1]. Although the mortality rate among older people in China is decreasing, their physical and cognitive functions are also decreasing [2]. This means older people in China need more long-term care services, and these services are facing greater challenges. Therefore, since 2016, China has implemented a long-term care insurance (LTCI) policy to relieve the problems created by population aging and associated issues in the healthcare system [3]. The LTCI is a pre-plot social insurance system among 15 selected cities in China, which provides basic life care and daily nursing services to the disabled and older adults, and also shares the nursing expenses incurred by people who cannot take care of themselves due to chronic diseases or physical and psychological disability [3, 4]. Target population and services of the LTCI are different among pilot cities. In Shanghai, employees and residents (including both urban and rural), who covered by the urban employee basic medical insurance scheme (UEBMI), the urban resident basic medical insurance scheme (URBMI), and the new rural cooperative medical system (NRCMS), respectively, are eligible to participate in the LTCI. However, urban and rural residents must be aged ≥60 years, while there is no such age requirement for employees. The Shanghai LTCI provides benefits mainly via institutional care services, home care services, and community care services [5], which not only includes basic life help but also health care and psychological comfort [6].

The World Report on Ageing and Health defined healthy aging as the process of maintaining and developing functional ability that enables well-being among older people [7]. Therefore, the most important consideration for an older person is preventing and reducing disability. Disability is commonly defined as difficulty experienced in activities, such as basic activities of daily living (BADL) and instrumental activities of daily living (IADL) [8]. Limitations in BADL or IADL reduce the ability of older people to live independently, and decrease their quality of life [9]. A 5-year follow-up study in Japan showed that patients aged over 65 years with low activities of daily living (ADL) scores had twice the mortality rate of patients with high scores [10]. Advanced age [11], being female [12], having chronic diseases, low engagement in physical activity, lack of social contacts, and poor self-rated health have been associated with the incidence of disability [13, 14]. In addition, multimorbidity accelerates the decline of functional ability among older people. As the number of chronic diseases increases, a person’s disability becomes more serious [15], which was also demonstrated by a Chinese community-based study [16].

Multimorbidity is commonly defined as the simultaneous occurrence of two or more chronic diseases in an individual [17]. A previous study considered the co-occurrence of diseases in individuals and evaluated the cumulative effect of chronic diseases based on multimorbidity [18]. Multimorbidity presents a major challenge for healthcare systems [19] and is becoming more common among older people as longevity increases [20, 21]. A prospective study in Europe found that more than 50% of older people reported having two or more diseases [22]. Similarly, a cross-sectional survey conducted in Southern China showed that 47.5% of older adults suffered from multiple diseases [23]. Compared with older people with a single chronic condition, those with multimorbidity have an increased risk for becoming dependent on long-term care [24]. The major consequences of multimorbidity include functional disability, poor quality of life, high mortality risk, and high healthcare utilization and costs [18]. Rizzuto and colleagues [25] found that older people with multiple diseases experienced disability in 81% of the remaining years of their life. A meta-analysis also suggested that the mortality rate in older people with multimorbidity was 44% higher than those with no or only one chronic disease [26]. Most available studies on this topic have focused on the relationship between the number of chronic diseases and disability in older people [27, 28]. However, specific combinations of diseases may have different effects on older people’s functional ability [29]. Yokota and colleagues [30] found that the most important factors contributing to the burden of disability were musculoskeletal, cardiovascular, and chronic respiratory diseases. Cardiovascular diseases were associated with greatest decline in BADL, and neurological/mental diseases were significantly associated with decline in IADL [31]. Chronic diseases with the same pattern may also share common risk factors and pathophysiological characteristics [32]. Therefore, researching multimorbidity patterns could offer a comprehensive understanding of the relationship between chronic diseases and functional ability among older people.

Clarifying the association between multimorbidity and disability is important for formulating long-term care policies and strategies, reducing functional disability, and improving the health-related quality of life among older people. This study aimed to explore the relationship between multimorbidity patterns and BADL/IADL disability among older people covered by LTCI in Shanghai, China.

## Methods

### Study population and data collection

This cross-sectional survey was part of broader research on improving the Shanghai LTCI that was conducted in 2018. Shanghai is located in Eastern China and divided into 17 districts, grouped into a central urban area (eight districts), suburbs (five districts), and outer suburbs (four districts) [33]. The distribution of the population of older people is uneven and there are environmental, social, and economic differences among these districts; therefore, we used multi-stage stratified cluster random sampling to enroll participants. At the first stage, we selected three districts from the central urban area, two from the suburbs, and one from the outer suburbs in Shanghai (Xuhui, Putuo, Jingan, Baoshan, Songjiang, and Jinshan districts). Then, in each selected district, one street community was selected randomly, and a total of 6 street communities were selected. Next, 1–3 committees were randomly selected from each selected street community. Lastly, older people aged ≥60 years and also covered by LTCI were randomly recruited as participants in this study. Data were collected using questionnaires completed through face-to-face interviews. The interviews were conducted by specially trained investigators who visited participants’ homes or facilities. The participants received a verbal description of the purposes and procedures of the study and informed consent is needed before the interview. Individuals who were not able to carry out proper verbal communication, due to being deaf or mute and dementia or cognitive impairments, were excluded. In total, 1871 older people were eligible for analysis. This study was approved by the Fudan University Research Ethics Committee. The study protocol was performed in accordance with the relevant guidelines.

### Measurement

#### Chronic diseases and multimorbidity

We identified chronic diseases through participants’ self-report and an assessment scale. Based on the National Health Service Surveys (NHSSs) in China and the physical health section of the Older Americans Resources and Services (ORAS) [34], we identified 33 chronic diseases by asking participants: “Have you been diagnosed with the following diseases by a doctor in the past 6 months?” The listed diseases were: hypertension, diabetes, cardiovascular disease (heart attack and other cardiovascular disorders), cerebrovascular disease, bronchitis, pneumonia, emphysema, asthma or chronic obstructive pulmonary disease, tuberculosis, cataract, glaucoma, cancer, prostatitis/prostatic hypertrophy, Parkinson disease, bedsores, injury or poisoning, rheumatoid arthritis, intervertebral disc disease, chronic low back pain, dyslipidemia, severe vision loss, psychiatric disease, lower extremity varicose veins, gout, hemorrhoids, hypothyroidism, non-inflammatory gynecological diseases, psoriasis, osteoporosis, chronic cholecystitis/gallstones, urinary stones, and anemia. In addition, participants’ depression was assessed by the 30-item Geriatric Depression Scale (GDS-30). Participants respond to each item with “yes” or “no.” The total score ranges from 0 to 30, with higher scores indicating greater depressive symptoms. We used a cut-off score of > 10 to define older people with possible depressive symptoms [35]. The GDS-30 appears to be an effective screening instrument for depression symptoms among Chinese older people [36].

#### Functional disability

Functional disability was assessed by asking older people about BADL and IADL. BADL disability was measured using the Barthel Index, which covers eating, bathing, grooming, dressing, bowel control, urination control, toileting, bed and chair transfer, walking on the ground, and ascending/descending stairs [37]. Based on the final score, participants’ degree of dependence was classified into four groups: severe ≤40, moderate = 41–60, mild = 61–99, and no dependence = 100. IADL disability was assessed with the Lawton IADL Scale, which covers using telephones, shopping, food preparation, housekeeping, doing laundry, using transportation, taking medications, and managing money [38]. The total score ranges from 0 to 8. Based on the total score, participants were classified as severe ≤2, moderate = 3–5, mild = 6–7, or no dependence = 8. There were only 190 and 46 participants who had no dependence in BADL and IADL, respectively, in order to ensure statistical validity, we considered severe and moderate dependence as disability, and mild and no dependence as independent/no disability.

### Covariates

Sociodemographic characteristics included care model (home, facility), sex (male, female), age (60–69, 70–79, 80–89, and ≥ 90 years), education level (illiterate, primary school, middle school, junior college or above), marital status (married, single [unmarried, divorced, widowed]), monthly income (< 1800, 1800–3499, 3500–3999, and ≥ 4000 CNY) [39]. Social support was assessed using the Perceived Social Support Scale [40]. This 12-item scale measures the level of social support from family, friends, and others. The total score ranges from 12 to 84, with higher scores indicating a higher level of social support. A score < 32 indicates a severe deficiency in social support, 32–49 reflects a low level of social support, and ≥ 50 indicates high social support [41].

### Statistical analysis

We performed descriptive analyses using frequencies (percentage) for categorical variables. Exploratory factor analysis was used to identity multimorbidity patterns. Each chronic disease was coded as a dichotomous variable (0 = no disease, 1 = disease present). Therefore, we used the principal factor method based on a tetrachoric correlation matrix [42]. The Kaiser-Meyer-Olkin method and Bartlett’s test of sphericity were used to estimate the adequacy of the data. Eigenvalues greater than 1 and the shape of the scree plot were used to determine the number of retained factors [43]. We used an oblique rotation (Oblimin) for factor loading matrices to obtain a better interpretation. The resulting factor loadings represented the strength of the association between the condition and the latent factor. We excluded conditions with a prevalence < 5.0% to ensure the results were robust [44]. Chronic disease was included in a given multimorbidity pattern if its loading was ≥0.3 in that pattern [31]. When the factor loading was ≥0.3 in more than one factor, that disease was assigned to the group with the largest loading value.

Binary logistic regression models were used to examine the association between the number and patterns of multimorbidity and BADL/IADL disability. All models were adjusted for care model, sex, age, education level, marital status, income, and social support. We used SPSS version 25.0 for all statistical analyses. *P*-values < 0.05 were considered statistically significant.

## Results

### Participants’ characteristics

This study included 1871 participants aged ≥60 years. The mean age was 83.6 years and the median/IQR was 85 (79, 89). 61.0% of all participants were female. We found that 57.0% of participants lived in their own homes, 57.7% were married, and 55.4% had a higher monthly income (≥ 3500). About one-third of the participants reported they were illiterate, but the majority had high social support. The prevalence of multimorbidity was 74.3%. Overall, the incidence rate of BADL disability and IADL disability were 50.7 and 90.7% respectively. Participants who received care in facilities had advanced age, and had lower social support level were more likely to have higher prevalence of BADL and IADL disability (Table 1).

**Table 1:** Participants’ characteristics at baseline and the prevalence of disability (*N* = 1871)

Characteristics	N (%)	BADL disability	IADL disability
Total Care model	1871 (100.0)	949 (50.7)	1696 (90.7)
Home	1067 (57.0)	510 (47.8)	948 (88.9)
Facility	804 (43.0)	439 (54.6)	748 (93.0)
Sex			
Male	729 (39.0)	395 (54.2)	663 (91.0)
Female	1142 (61.0)	554 (48.5)	1033 (90.5)
Age, years			
60–69	123 (6.6)	67 (54.5)	111 (90.2)
70–79	345 (18.4)	160 (46.4)	305 (88.4)
80–89	1009 (53.9)	478 (47.4)	897 (88.9)
≥ 90	394 (21.1)	244 (61.9)	383 (97.2)
Education level			
Illiteracy	656 (35.1)	300 (45.7)	591 (90.1)
Primary school	473 (25.3)	279 (59.0)	437 (92.4)
Middle school	574 (30.7)	288 (50.2)	518 (90.2)
Junior college or above	168 (9.0)	82 (48.8)	150 (89.3)
Marital status			
Single	791 (42.3)	405 (51.2)	715 (90.4)
Married	1080 (57.7)	544 (50.4)	981 (90.8)
Income, CNY^a^			
<1800	460 (24.6)	206 (44.8)	414 (90.0)
1800–3499	375 (20.0)	184 (49.1)	344 (91.7)
3500–3999	239 (12.8)	130 (54.4)	214 (89.5)
≥ 4000	797 (42.6)	409 (51.3)	724 (90.8)
Social support			
Very low	58 (3.1)	43 (74.1)	54 (93.1)
Low	706 (37.7)	392 (55.5)	646 (91.5)
High	1107 (59.2)	514 (46.4)	996 (90.0)
Multimorbidity			
No	481 (25.7)	239 (49.7)	415 (86.3)
Yes	1390 (74.3)	710 (51.1)	1281 (92.2)

^a^*CNY* Chinese Yuan,1 US $ equals about CNY 6.56

*BADL* Basic activities of daily living; *IADL* Instrumental activities of daily living

Severe and moderate dependence = disability, mild and no dependence = independent/ no disability

Categorical variables are presented as number (%)

**Fig. 1:**
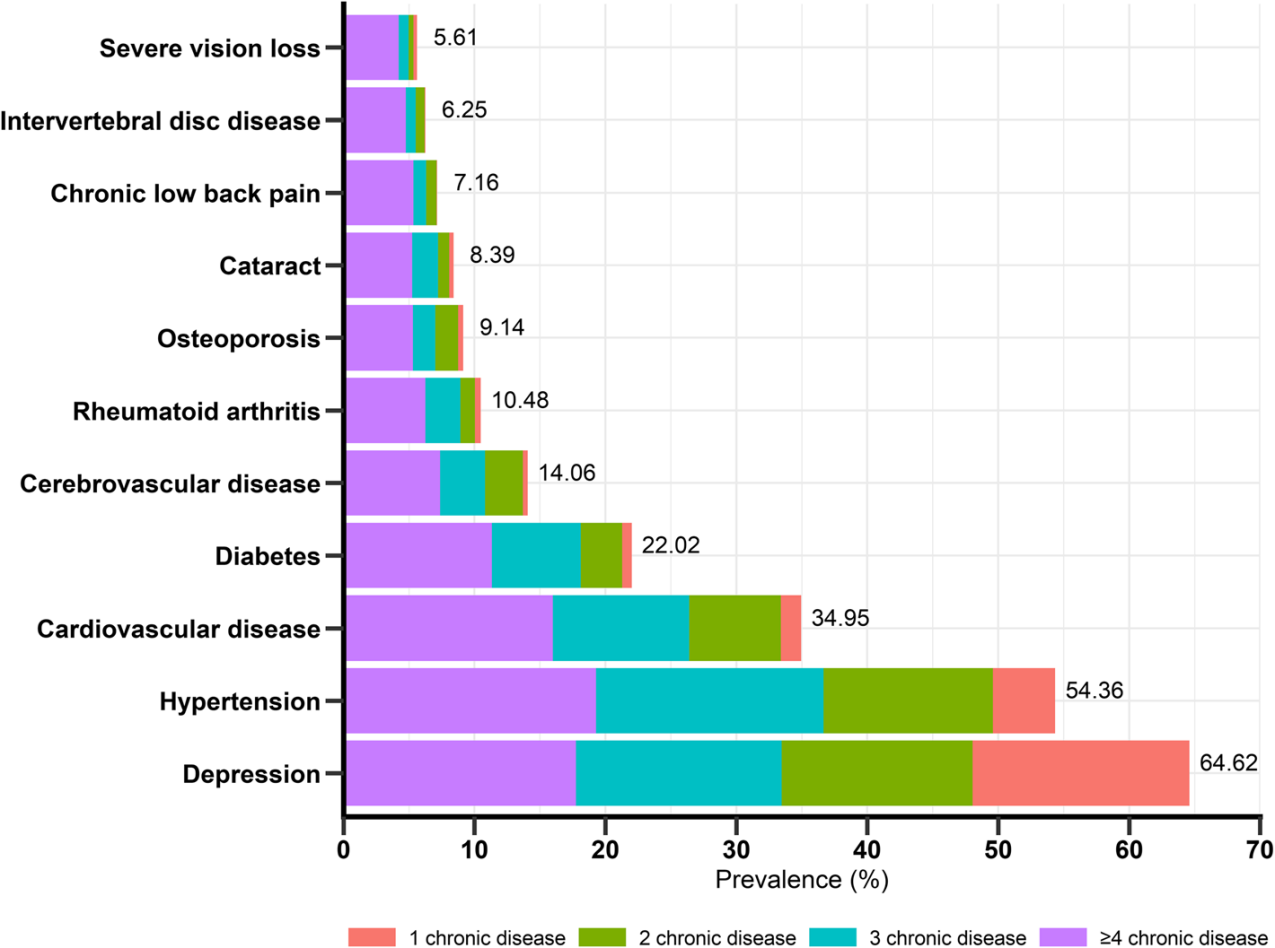
Prevalence (per 100 population) and proportions of multimorbidity (*N* = 1871)

Figure 1 shows the prevalence and proportions of multimorbidity for the 11 chronic diseases with a prevalence > 5.0% and thus included in the analysis. The most common disease was depression (64.62%), followed by hypertension (54.36%) and cardiovascular disease (34.95%). Diabetes, cerebrovascular disease, and rheumatoid arthritis showed prevalence rates of 10.5–22.0%, whereas the other disorders were less frequently reported (≤ 10.0%).

### Factor analysis results

Of the 11 conditions included in the factor analysis, we identified three multimorbidity patterns that explained 41.6% of the variance (The scree plot is in the Additional File 1). The first pattern was a musculoskeletal disease pattern that comprised rheumatoid arthritis, intervertebral disc disease, and chronic low back pain. The second pattern was a cardio-metabolic disease pattern that was characterized by hypertension, diabetes, cardiovascular disease, and cerebrovascular disease. Finally, the third pattern was a mental-degenerative disease pattern that included cataracts, severe vision loss, osteoporosis, and depression (Table 2).

**Table 2:** Rotated factor loadings (Oblimin) for 11 chronic diseases by disease patterns from the factor analysis

Chronic Diseases	The musculoskeletal disease pattern	The cardio-metabolic disease pattern	The mental-degenerative disease pattern
Hypertension	−0.01	**0.70**	−0.10
Diabetes	−0.08	**0.61**	−0.03
Cardiovascular disease	0.02	**0.59**	−0.11
Cerebrovascular disease	0.08	**0.40**	0.23
Rheumatoid arthritis	**0.46**	−0.15	0.06
Intervertebral disc disease	**0.82**	−0.11	0.05
Chronic low back pain	**0.79**	−0.08	0.07
Cataract	−0.04	0.25	**−0.65**
Severe vision loss	0.17	0.18	**−0.55**
Osteoporosis	0.40	−0.02	**−0.40**
Depression	−0.10	0.29	**0.56**

Bold numbers indicate the highest factor loading for each chronic disease

### Chronic diseases and BADL/IADL disability

Table 3 shows the prevalence of multimorbidity and the associations with BADL/IADL disability. We found that 5.8% of participants had no chronic diseases, 19.9% had one disease, 18.6% had two diseases, 21.2% had three diseases, and 34.5% had four or more chronic diseases. The average number of chronic diseases per participant was 3.0 ± 2.0. Having multimorbidity was significantly associated with functional disability. After adjusting for care model, sex, age, education level, marital status, income, and social support, we found that four groups were significantly associated with BADL disability: one disease (OR 2.73, 95%CI 1.70–4.38), two diseases (OR 1.74, 95%CI 1.08–2.80), three diseases (OR 2.15, 95%CI 1.35–3.45), and four or more diseases (OR 3.38, 95%CI 2.15–5.32). In addition, three groups were significantly associated with IADL disability: two diseases (OR 1.97, 95%CI 1.07–3.61), three diseases (OR 2.52, 95%CI 1.37–4.63), and four or more diseases (OR 5.44, 95%CI 2.91–10.16).

**Table 3:** Associations between multimorbidity and functional disability (*N* = 1871)

No. Of chronic conditions	Prevalence, n (%)	BADL disability	IADL disability
		OR (95% CI)^a^	OR (95% CI)^a^
0	108 (5.8)	Reference	Reference
1	373 (19.9)	**2.73 (1.70–4.38)**	1.69 (0.93–3.06)
2	348 (18.6)	**1.74 (1.08–2.80)**	**1.97 (1.07–3.61)**
3	397 (21.2)	**2.15 (1.35–3.45)**	**2.52 (1.37–4.63)**
≥4	645 (34.5)	**3.38 (2.15–5.32)**	**5.44 (2.91–10.16)**

*BADL* Basic activities of daily living; *IADL* Instrumental activities of daily living

Severe and moderate dependence = disability, mild and no dependence = independent/ no disability

*OR* Odd ratio, *IC* Confidence interval

Boldface indicates statistical significance (*p* < 0.05)

^a^All models were adjusted for care model, sex, age, education level, marital status, income, and social support

Table 4 shows the results of the binary logistic regression model investigating the associations between multimorbidity patterns and BADL/IADL disability. After adjusting for care model, sex, age, education level, marital status, income, and social support, we found that the cardio-metabolic disease pattern was associated with both BADL (OR 1.28, 95%CI 1.16–1.41) and IADL (OR 1.41, 95%CI 1.19–1.68) disability, and the mental-degenerative disease pattern was associated with BADL disability (OR 1.55, 95%CI 1.40–1.72).

**Table 4:** Associations between multimorbidity patterns and BADL and IADL disability (*N* = 1871)

Multimorbidity patterns	BADL disability		IADL disability	
	Unadjusted OR (95% CI)	Adjusted OR (95% CI)^a^	Unadjusted OR (95% CI)	Adjusted OR (95% CI)^a^
Musculoskeletal diseases	0.93 (0.85–1.03)	0.94 (0.85–1.03)	1.12 (0.93–1.35)	1.15 (0.95–1.38)
Cardio-metabolic diseases	**1.24 (1.13–1.37)**	**1.28 (1.16–1.41)**	**1.32 (1.12–1.56)**	**1.41 (1.19–1.68)**
Mental-degenerative diseases	**1.53 (1.38–1.69)**	**1.55 (1.40–1.72)**	1.15 (0.99–1.35)	1.17 (0.99–1.37)

*BADL* Basic activities of daily living; *IADL* Instrumental activities of daily living

Severe and moderate dependence = disability, mild and no dependence = independent/ no disability

*OR* Odd ratio, *IC* Confidence interval

Boldface indicates statistical significance (*p* < 0.05)

^a^Adjusted for care model, sex, age, education level, marital status, income, and social support

## Discussion

This cross-sectional study showed multimorbidity and disability were highly prevalent among older people covered by LTCI in Shanghai. Both the number and pattern of chronic diseases were associated with functional disability, but had different effects on BADL and IADL disability. This suggested that specific combinations of chronic diseases may be useful in predicting functional disability. Understanding these patterns is important to help prevent functional disability and inform the development of long-term care policies. To our knowledge, this was the first population-based study among older people covered by LTCI in China that investigated the relationship between multimorbidity patterns and functional ability.

The prevalence of multimorbidity in this study was 74.3%, which was consistent with previous examinations both locally and abroad. Internationally, the prevalence of multimorbidity among older people ranges from 55.0 to 98.0% [18]. And the prevalence of multimorbidity among people aged over 60 years in communities in China ranges from 6.4 to 76.5% [45]. These findings suggest that multimorbidity is a global problem. Among these chronic diseases, depression is the chronic disease with the highest prevalence (64.62%). To our knowledge, current findings about the depressive symptoms among older people living at long-term care institutions were mixed. For example, close to our results, an observational study in Poland also found that the prevalence of depression among older individuals living in long-term care institutions was 56.9% [46]. Mansbach et al. [47] used the Brief Anxiety and Depression Scale to assess the prevalence of depression among long-term care residents in America, and the result showed that the prevalence was 54.5%. However, another study conducted in Rhode Island, USA suggested that only 28.6% of all residents in long-term care facilities suffered from depression [48]. Wongpakaran et al. [49] found that the prevalence of depression among institution-dwelling older adults was 23.5% in northern Thailand. Some reasons could be possible explanation for this. First, different measurement tools were used to assess the depressive symptoms among these studies. Another possible reason for this difference may be that the proportion of the oldest-old (aged ≥80 years) was high in the present study, and previous conclusion has determined that the risk of depression increased with advanced age [50].

Our study also showed there was a high prevalence of functional limitations among older people. The percentage of older people with disability in BADL was 50.7%, and that of IADL disability was 90.6%. These percentages were higher than those observed in a previous community-based study in Shanghai that reported 23.2% of older adults had ADL disability and 37.9% had IADL disability [16]. However, the discrepancies between the studies may be attributable to the fact that in our study, participants were covered by LTCI, and most had some self-care disability and low health status.

This study revealed there was a significant association between the number of chronic diseases and the likelihood of BADL disability and IADL disability among older people, which was consistent with previous studies [51, 52]. Arokiasamy et al. [53] showed that the association between chronic conditions and functional ability was stronger as the number of chronic conditions increased. Unfortunately, that study did not evaluate IADL items and the risk for IADL decline associated with the number of chronic conditions was not clearly described. Our study included eight IADL items, and comprehensively assessed the association between the number of chronic diseases and functional ability. The findings highlight the need for further investigations to explore whether interventions to reduce multimorbidity may help maintain and improve functional ability among older people.

This study identified three multimorbidity patterns: the musculoskeletal disease pattern, the cardio-metabolic disease pattern, and the mental-degenerative disease pattern. Differences in multimorbidity patterns across studies may be partly attributable to differences in participants’ characteristics, and the composition and assessment approaches of chronic conditions. However, previous authors concluded that at least three broad patterns were reflecting cardiovascular and metabolic diseases, mental problems, and musculoskeletal disorders [32], which was similar to our findings. Kirchberger et al. [54] identified that the cardiovascular/metabolic disorder pattern was characterized by hypertension, heart diseases, diabetes, and stroke, which was also consistent with our study. In addition, other studies have found that the musculoskeletal disease pattern included arthritis, back pain, and other chronic pain [55]. Finally, a Chinese rural study also classified mental and degenerative diseases in the same pattern [56], and another study reported an association between degenerative disorders and depression symptoms [57]. We consider that these findings support the mental-degenerative disease pattern identified in our study.

We showed that the cardio-metabolic disease pattern and the mental-degenerative disease pattern were associated with functional ability, with the cardio-metabolic disease pattern being associated with both BADL and IADL disability. A longitudinal study showed that the cardiovascular disease pattern was associated with the greatest decline in ADL compared with other patterns [31]. The large contribution of diseases in this pattern to functional limitations may be associated with modifiable lifestyle risk factors, such as obesity, smoking, alcohol consumption, and physical inactivity [58]. This highlights the need for interventions focused on lifestyle management among older people. We also found that the mental-degenerative disease pattern was significantly associated with functional disability. Previous studies found that both mental disorders and degenerative diseases were negatively associated with functional performance among older people [59, 60]. Moreover, combinations of somatic and mental disorders have been associated with a greater incidence of disability than combinations of somatic conditions only [61]. This suggested that more attention should be directed to properly managing physical and mental diseases among older people to maintain their functional ability. We found no significant association between the musculoskeletal disease pattern and functional ability, which was consistent with a longitudinal Australian study among older females [31]. However, previous studies reported that musculoskeletal disorders made a major contribution to the disability burden [62, 63]. A community-based study from Poland found that more than two-thirds of people with musculoskeletal disorders also suffered from mobility problems, including difficulty standing or walking for long distances [59]. In addition, that study indicated more than 90% of these people experienced limitations in participating in social life. However, it is possible that a considerable proportion of older people in our study received drug or clinical therapy that minimized any pain symptoms. Therefore, older people in our sample might not have reported musculoskeletal disorders. Moreover, this may be due to the type of diseases included in our study. Musculoskeletal disorders consist of multiple diseases, but only rheumatoid arthritis, intervertebral disc disease, and chronic low back pain are included in the musculoskeletal disease pattern. Besides, a possible explanation could be that Shanghai is one of the most prosperous cities in China, rapid urbanization and economic growth benefit people to reduce the incidence of physical disorders. In line with our results, a survey in eight European countries also showed that self-reported musculoskeletal conditions were negatively related to socio-economic status [64]. Our findings indicate that it is necessary to develop targeted prevention and healthcare strategies that consider patterns of chronic conditions among older people.

This study suggests that multimorbidity may be associated with disability. Although the majority of previous studies support the assumption that chronic conditions cause functional impairment [65, 66], other studies have suggested that there is an interaction between multimorbidity and functional decline [67]. On one hand, disorders classified in common patterns of multimorbidity may interact, which could curtail compensatory mechanisms and accelerate functional decline. On the other hand, functional disability may affect people’s illness and treatment burden and their response capacity, which may further increase multimorbidity and contribute to establishing a vicious circle. In addition, a synergistic effect of multimorbidity and disability has been reported in a study conducted in the United States [68]. That study showed that older people with both multimorbidity and functional limitations used healthcare resources more frequently and intensively than those with multimorbidity or functional limitation only. Therefore, more attention should be paid to older people with both multimorbidity and functional disability.

The use of factor analysis to identify multimorbidity patterns has various strengths. For example, it facilitates a better understanding of how conditions (as opposed to individuals) are naturally grouped. Moreover, our population-based study evaluated a range of chronic diseases that impact older people’s health status. However, our study had some limitations. First, causality between chronic multimorbidity and disability cannot be determined with cross-sectional data. Therefore, further studies with prospective designs are needed to examine causality and consider the interaction and synergistic effect of multimorbidity and disability. Second, a potentially important problem is that some chronic conditions were collected based on participants’ self-reported information. Reporting diseases not only depends on the actual presence of clinical conditions but also depends upon the characteristics of participants. Such as knowledge and understanding about problems, the consequence of chronic conditions for daily life, willingness of reporting, and their frequency of contact with physicians [69–71]. Therefore, these data about chronic diseases may be susceptible to inaccuracy and we might underestimate their prevalence. Similarly, memory tends to decline with advanced age, older people might forget or misremember their chronic conditions/diseases. Hence, the possibility of recall bias could not be excluded in this study. Third, detailed information regarding disease severity was not collected in this study, which might have affected the results. Further research is necessary that considers disease severity.

## Conclusions

We found that multimorbidity and functional disability were highly prevalent among older people covered by LTCI in Shanghai, China. In addition, multimorbidity has specific patterns (i.e., cardio-metabolic and mental-degenerative disease patterns) that are associated with functional disability among older people. The present findings offer new insights regarding the association between chronic diseases and functional disability in this population. This information may inform the design of appropriate long-term healthcare and preventative strategies to reduce chronic diseases, prevent functional disability, and improve the health-related quality of life among older people. It also important for integrating care programs for multimorbidity. Further longitudinal research is needed to investigate the causality between multimorbidity patterns, disease severity, and disability.

## Supplementary Information

**Additional file 1.** The scree plot of the factor analysis, determining the number of retained factors.

## Data Availability

The datasets generated during and/or analyzed during the current study are available from the corresponding author on reasonable request.

## Abbreviations

BADL: Basic activities of daily living
IADL: Instrumental activities of daily living
IC: Confidence interval
OR: Odds ratio
