# Supplementary figures and images for: Association between multimorbidity patterns and disability among older people covered by long-term care insurance in Shanghai, China
Source: BMC Public Health. 2021 Feb 27;21:418. doi: 10.1186/s12889-021-10463-y (PMC7912511; doi:10.1186/s12889-021-10463-y)

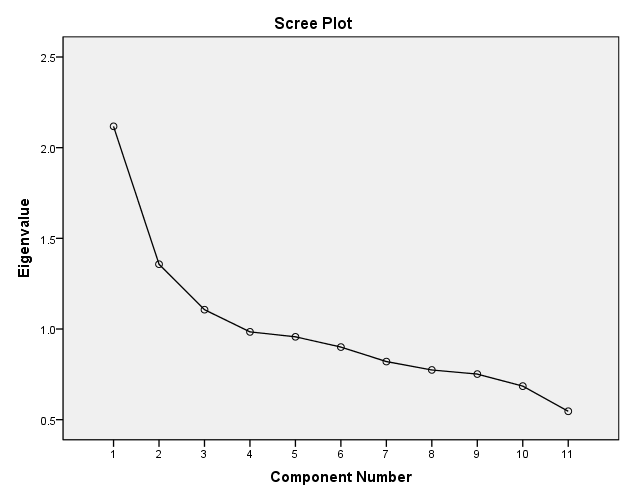


**Figure S1.** The scree plot of factor analysis

Supplement: Supplementary file 1 — Additional file 1. The scree plot of the factor analysis, determining the number of retained factors. [file 12889_2021_10463_MOESM1_ESM.docx]
